# Evaluating the understanding of the ethical and moral challenges of Big Data and AI among Jordanian medical students, physicians in training, and senior practitioners: a cross-sectional study

**DOI:** 10.1186/s12910-024-01008-0

**Published:** 2024-02-17

**Authors:** Abdallah Al-Ani, Abdallah Rayyan, Ahmad Maswadeh, Hala Sultan, Ahmed Alhammouri, Hadeel Asfour, Tariq Alrawajih, Sarah Al Sharie, Fahed Al Karmi, Ahmed Mahmoud Al-Azzam, Asem Mansour, Maysa Al-Hussaini

**Affiliations:** 1 Office of Scientific Affairs and Research, King Hussein Cancer Center, Amman, Jordan; 2 Faculty of Medicine, University of Jordan, Amman, Jordan; 3 Faculty of Medicine, Yarmouk University, Irbid, Jordan; 4 Office of Director General, King Hussein Cancer Center, Amman, Jordan; 5 Department of Pathology and Laboratory Medicine, King Hussein Cancer Center, 202 Queen Rania Street, Amman, 11941 Jordan

**Keywords:** Ethics, Artificial intelligence, Big data, Ownership, Privacy, Bias, Epistemology, Accountability, Jordan, Medical students

## Abstract

**Aims:**

To examine the understanding of the ethical dilemmas associated with Big Data and artificial intelligence (AI) among Jordanian medical students, physicians in training, and senior practitioners.

**Methods:**

We implemented a literature-validated questionnaire to examine the knowledge, attitudes, and practices of the target population during the period between April and August 2023. Themes of ethical debate included privacy breaches, consent, ownership, augmented biases, epistemology, and accountability. Participants’ responses were showcased using descriptive statistics and compared between groups using t-test or ANOVA.

**Results:**

We included 466 participants. The greater majority of respondents were interns and residents (50.2%), followed by medical students (38.0%). Most participants were affiliated with university institutions (62.4%). In terms of privacy, participants acknowledged that Big Data and AI were susceptible to privacy breaches (39.3%); however, 59.0% found such breaches justifiable under certain conditions. For ethical debacles involving informed consent, 41.6% and 44.6% were aware that obtaining informed consent posed an ethical limitation in Big Data and AI applications and denounced the concept of “broad consent”, respectively. In terms of ownership, 49.6% acknowledged that data cannot be owned yet accepted that institutions could hold a quasi-control of such data (59.0%). Less than 50% of participants were aware of Big Data and AI’s abilities to augment or create new biases in healthcare. Furthermore, participants agreed that researchers, institutions, and legislative bodies were responsible for ensuring the ethical implementation of Big Data and AI. Finally, while demonstrating limited experience with using such technology, participants generally had positive views of the role of Big Data and AI in complementing healthcare.

**Conclusion:**

Jordanian medical students, physicians in training and senior practitioners have limited awareness of the ethical risks associated with Big Data and AI. Institutions are responsible for raising awareness, especially with the upsurge of such technology.

**Supplementary Information:**

The online version contains supplementary material available at 10.1186/s12910-024-01008-0.

## Introduction

During recent years, there has been a surge of Big Data usage within healthcare. Nonetheless, the scientific community has yet to reach a concrete definition of what Big Data really is [1], as generic definitions use Big Data whenever traditional modes of computational storage or analysis are not sufficient to deal with large and dynamic datasets. Within the context of this paper, Big Data refers to “analytics that can process massive quantities of data in the search for information, including unforeseen information” and is characterized by the three V’s (i.e., volume (huge size), velocity (created in near real-time), and variety (diversity of content)) [2]. Other scholars also employ versatility, volatility, virtuosity, vitality, and vibrancy among other concepts to the definition of Big Data; however, that only serves to muddle the definition of Big Data which is barely consistent across its applications [3].

Today’s Big Data culture embraces cyber-physical systems, cloud computing, and the Internet of Things [4]; all of which are a pertinent component of Industry 4.0. Such a culture, by necessity, aspires for the digitization, datafication, and networking of any objects within its networks [5]. This has led to a number of high-profile cases which have raised concern about the moral and legal aspects of such technology [6]. Such cases include Cambridge Analytica’s targeting of American voters in 2016, YouTube extracting children’s personal information without consent for advertisement targeting, and DeepMind Technologies Ltd’s algorithm to assist in the management of acute kidney injury using a population-derived dataset with opaque regulation [7].

Similarly, artificial intelligence (AI), despite its raging popularity, is often as misunderstood by scientists as it is by the public. The current uses of AI can be traced to machine learning (ML), which simply put is dynamic algorithms trained on a large volume of data that are able to recognize patterns [8]. ML has also advanced into deep learning (DL), which refers to artificial networks created by algorithms for the purpose of “independent” decision-making. However, without extensive training, these systems are only black boxes of code dependent on a steady stream of large volumes of human data as to avoid overfitting [9].

Big Data, coupled with the rise of AI usage in diagnostics and decision-making across nearly every facet of medicine, shed light on the imperfections and lack of regulations with regard to modern analytics. Such issues include breach of privacy [10], consent [11], epistemological definitions [6], data ownership [5], methodological opacities [4, 12], and bias [13] to name a few. While Big Data and the AI system may smoothen diagnostic processes, enhance disease detection, establish quicker treatment, and anticipate patient outcomes [14, 15], their risks need to be acknowledged and regulated by those within healthcare.

Multiple reports throughout the literature demonstrate that healthcare workers across undergraduate and postgraduate levels demonstrate poor awareness of the most basic ethical principles when it comes to medical practice [16–20]. Therefore, it is expected that such a population may be liable to face further ethical dilemmas within the inherently complex context of Big Data and AI. Furthermore, recent literature on the perceptions of medical practitioners on Big Data and AI is superficial at best; often examining self-perceived understanding of AI-related definitions or mere attitudes towards the utility of AI [21–24]; which are often measured using inconsistent tools, leading to significant methodological heterogeneity. The aforementioned is further complicated by the lack of a comprehensive resource that complies with all the ethical challenges associated with such a technology.

In light of what’s above, this research aimed to explore the understanding of medical students and healthcare workers with regard to the ethical and moral challenges associated with Big Data and AI applications in medical practice. By providing a tool that encompasses the most pertinent and debated ethical dilemmas associated with such applications, this study seeks to establish a baseline assimilation of the prevalent views and awareness gaps among medical practitioners toward AI. Furthermore, this study strives to shed light on the attitudes and practices of Jordanian medical students and healthcare workers toward such applications. Finally, the study also seeks to determine if there are institutional differences in the perceptions of ethical challenges associated with Big Data and AI.

## Methodology

### Study design and setting

Using a cross-sectional design, this study used a self-administered questionnaire to assess the knowledge, attitudes, and practices (KAP) of healthcare workers towards the ethics of AI and Big Data between April and August, 2023. Healthcare personnel were recruited from all of Jordan’s medical establishments including university (e.g., Jordan University Hospital), private (e.g., Istishari Hospital), public (e.g., Al-Bashir Hospital), military (e.g., Royal Medical Services), and specialized hospitals (King Hussein Cancer Center).

### Participants’ characteristics

Participants working within any healthcare field and who had consented to participate were included in the final analysis. Participants were categorized per their level of training into undergraduates (i.e., medical students), postgraduate physicians in training (i.e., interns and residents), and senior practitioners (i.e., fellows and specialists). Participants were approached through dedicated social media groups, WhatsApp groups, or face-to-face communication. Participants who did not complete at least 80% of the questionnaire were excluded. For online participants, a one-time completed policy was applied to avoid redundancy.

### Ethical considerations

This study was approved by the King Hussein Cancer Center Institutional Review Board and followed the institutional and/or national research committee’s ethical standards and the principles of the World Medical Association’s Declaration of Helsinki.

### Data collection instrument

The questionnaire was designed after a thorough and systematic literature review of articles pertaining to the ethical and moral dimensions of utilizing AI and Big Data technology. The questionnaire was created on and distributed through Google Forms. It is comprised of four distinct domains including 1) sociodemographics, 2) knowledge, 3) attitudes, and 4) practices. Included in socio-demographics were biological sex, educational level, number of publications, current institution, and familiarity with AI and Big Data applications at time of taking the questionnaire. The knowledge domain addressed a variety of sub-themes including a) privacy and confidentiality, b) informed consent, c) data ownership, d) biases and divides created by AI and Big Data, e) epistemology, and f) accountability. Questions related to the knowledge, attitudes, and practices were scored using a 5-point Likert scale (1 = Strongly disagree, 2 = Disagree, 3 = Neutral, 4 = Agree, 5 = Strongly agree). After pilot testing on 30 senior doctors and 30 medical students, the Cronbach α for the knowledge, attitudes, and practices domains were 0.939, 0.898, and 0.747, respectively. The Cronbach α for the knowledge privacy and confidentiality, informed consent, ownership, biases and divides, epistemology, and accountability subdomains were 0.860, 0.772, 0.714, 0.827, 0.773, and 0.832, respectively. The questionnaire’s content validity index was 0.883.

The full questionnaire can be viewed as supplementary material (refer to Additional file 1).

### Sample size estimation

The estimated sample size was calculated using G*Power 3.1 and EpiInfo. At a power of 95%, α margin of error of 5% and an effect size of 50%, a sample of 220 participants was needed to demonstrate statistical differences of appropriate power.

### Statistical analysis

All data were organized, cleaned, and analyzed using SPSS version 23. Descriptive data was used to showcase the baseline characteristics and responses of included participants. Categorical data were shown as frequencies [n (%)], while continuous data were presented as medians and interquartile ranges. For items utilizing 5-point Likert scales, disagreement responses were grouped together (Strongly disagree and disagree into disagree), while agreement responses were grouped together for ease in reporting (strongly agree and agree into agree). Associations between categorical variables were examined using chi-square test. Mean differences in responses between categorical variables of two groups and more than two groups were examined using the t-test and ANOVA tests, respectively. All statistical tests are conducted with 95% confidence interval and 5% error margin. A *p*-value of 0.05 or less was considered statistically significant.

## Results

### Participants’ characteristics

A total of 466 participants responded to our survey. The included sample was characterized by a median age of 24.0 [22.0 – 26.0] and a male-to-female ratio of 1.04-to-1. Nearly 54.0% of respondents did not publish any peer-reviewed articles. Interns and residents comprised the greater majority of respondents (50.2%), followed by medical students (38.0%). Only 11.8% of respondents were clinical fellows or higher. The greater majority of respondents were affiliated with university institutions (62.4%). The King Hussein Cancer Center (KHCC) and Royal Medical Services (RMS) were the least represented (5.2% and 7.9%, respectively). In terms of familiarity with Big Data and AI applications in healthcare, the majority of respondents declared a moderate familiarity (34.5%). Table 1 shows the characteristics of the included participants.

**Table 1:** Characteristics of included participants

	**Total** **(*n* = 466)**	**Students** **(*n* = 177)**	**Interns & Residents** **(*n* = 234)**	**Fellows & Beyond** **(*n* = 55)**	***p*-value**
**Gender**					0.299
Female	228 (48.9%)	92 (52.0%)	114 (48.7%)	22 (40.0%)	
Male	238 (51.1%)	85 (48.0%)	120 (51.3%)	33 (60.0%)	
**Publication status**					** < 0.001**
Zero	251 (53.9%)	143 (80.8%)	96 (41.0%)	12 (21.8%)	
≥ 1	215 (46.1%)	34 (19.2%)	138 (59.0%)	43 (78.2%)	
**Current institution**					** < 0.001**
KHCC	24 (5.2%)	4 (2.3%)	9 (3.8%)	11 (20.0%)	
Private sector	72 (15.5%)	0 (0.0%)	50 (21.4%)	22 (40.0%)	
Public sector	42 (9.0%)	6 (3.4%)	35 (15.0%)	1 (1.8%)	
RMS	37 (7.9%)	0 (0.0%)	17 (7.3%)	20 (36.4%)	
University Hospitals	291 (62.4%)	167 (94.4%)	123 (52.6%)	1 (1.8%)	
**I am familiar with Big Data and AI applications in healthcare**					0.852
Strongly disagree	86 (18.5%)	37 (20.9%)	40 (17.1%)	9 (16.4%)	
Disagree	100 (21.5%)	31 (17.5%)	57 (24.4%)	12 (21.8%)	
Neutral	161 (34.5%)	61 (34.5%)	82 (35.0%)	18 (32.7%)	
Agree	75 (16.1%)	30 (16.9%)	35 (15.0%)	10 (18.2%)	
Strongly agree	44 (9.4%)	18 (10.2%)	20 (8.5%)	6 (10.9%)	

### Privacy & confidentiality

**Fig. 1:**
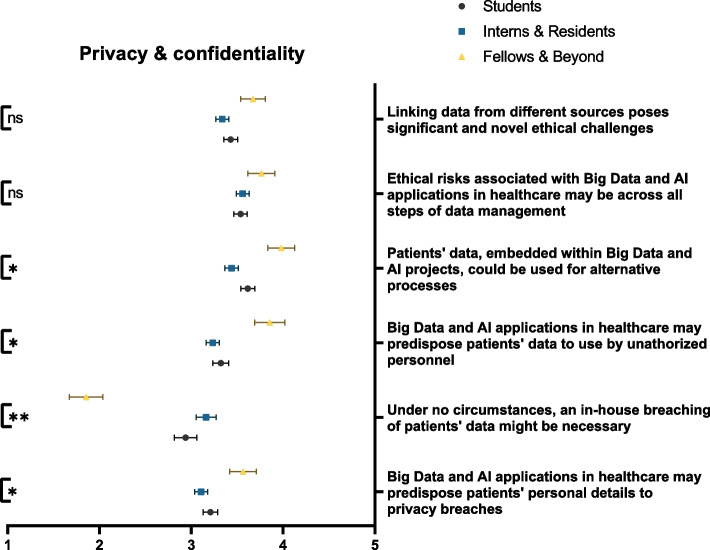
Participants’ awareness of the impact of Big Data and AI on privacy and confidentially, stratified by educational rank. ns: not significant; * *p* < 0.05; ** *p* < 0.001

Overall, respondents were fairly aware of the threats of Big Data and AI within healthcare. The greatest proportion of participants acknowledged that Big Data and AI may predispose patients’ data to privacy breaches (39.3%) or usage by unauthorized personnel (47.0%). Compared to students, interns, and residents, fellows and physicians of higher ranks were significantly more likely to acknowledge the aforementioned risks (*p* = 0.018 and *p* = 0.001, respectively). Respondents agreed that the ethical risks associated with Big Data and AI are significant and novel (47.6%) and may be present across all stages of data management (54.9%). Worryingly, only 41.0% concurred that an in-house breaching of patients’ data is never justified. The opposite notion was significantly accepted by physicians of higher ranks (*p* < 0.001) (Fig. 1). Analysis of participants’ responses stratified by gender, educational rank, and publication status is present in Additional file 2.

### Informed consent

**Fig. 2:**
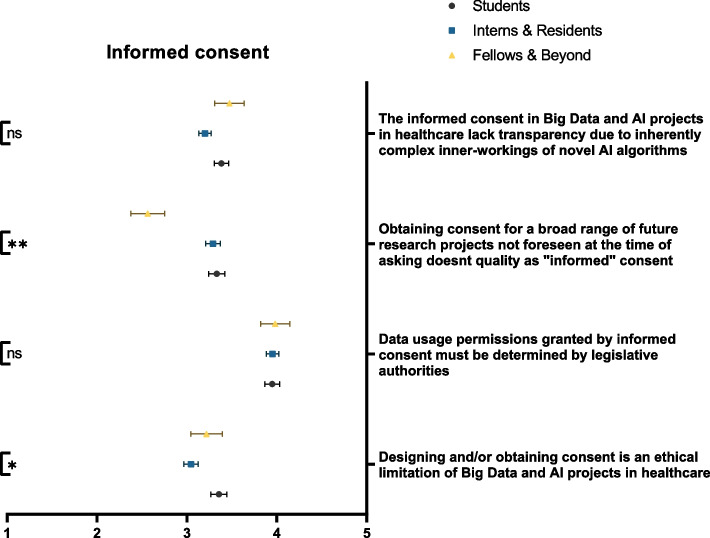
Participants’ awareness of the impact of Big Data and AI on informed consent, stratified by educational rank. ns: not significant; * *p* < 0.05; ** *p* < 0.001

A fair number of our respondents were able to distinguish the ethical dilemmas associated with informed consent. The majority of participants agreed that designing and/or obtaining consent is an ethical limitation in Big Data and AI applications in healthcare (41.6%). A notion that is significantly more appreciated by residents/interns than their student counterparts (*p* = 0.038). Similarly, 44.6% denounce the concept of ‘broad consent’ as it doesn’t qualify as informed consent; a statement that was significantly more rejected by senior physicians (*p* < 0.001) (Fig. 2). The greater majority of respondents (70.2%) believed that data usage permissions granted by informed consents should be regulated by legislative authorities. Interestingly, while 35.6% took neutral stances, 43.6% of respondents believed that informed consent in Big Data and AI could not be trusted due to the complex, and often concealed, inner-workings of AI algorithms.

### Ownership

**Fig. 3:**
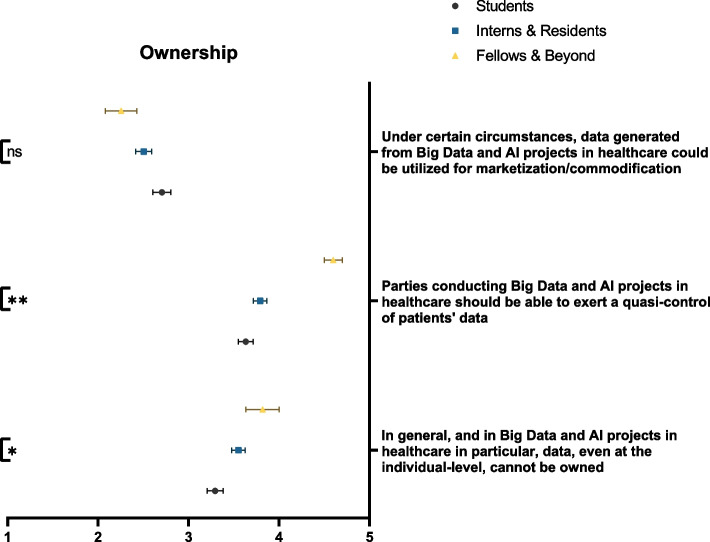
Participants’ awareness of the impact of Big Data and AI on data ownership, stratified by educational rank. ns: not significant; * *p* < 0.05; ** *p* < 0.001

In terms of data ownership, the majority of participants believed that data, irrespective of context, cannot be owned (49.6%). Such a fact was significantly more recognized by senior physicians than medical students (*p* = 0.008). However, they concurred that parties conducting Big Data and/or AI projects should be able to expert a quasi-control of patients’ data (59.0%), which was significantly more supported by fellows and senior physicians than students, interns, and residents (*p* < 0.001) (Fig. 3). Respondents also rejected the idea of using data generated from Big Data or AI projects for commodification purposes (49.6%).

### Biases & divides

**Fig. 4:**
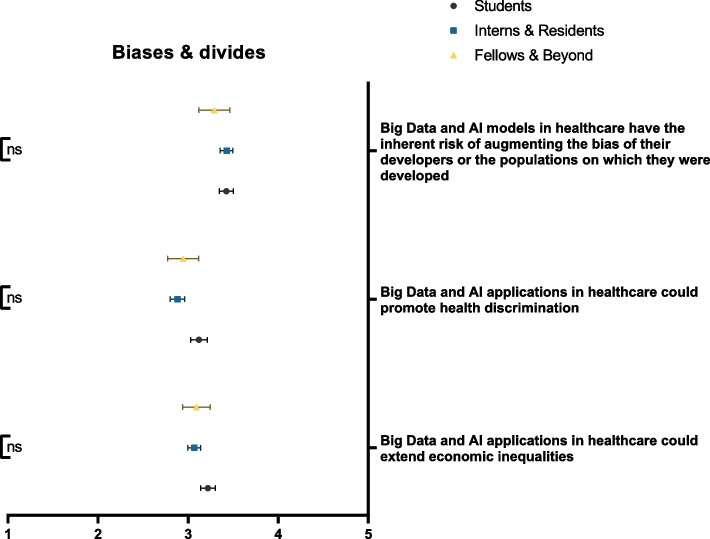
Participants’ awareness of the impact of Big Data and AI on augmenting or creating biases in healthcare, stratified by educational rank. ns: not significant; * *p* < 0.05; ** *p* < 0.001

When asked about the potential of Big Data and AI in promoting healthcare divides, there was no clear-cut census among respondents. Neutral stances were maintained for the effect of Big Data and AI on extending economic inequalities (40.8%) or promoting health discrimination (30.0%). Respondents believed that the aforementioned technologies could have inherent biases associated with either their developers or the populations upon which they were developed (49.1%). Figure 4 demonstrates participants’ responses to statements regarding AI-related biases and imbalances stratified by educational rank.

### Epistemology & accountability

**Fig. 5:**
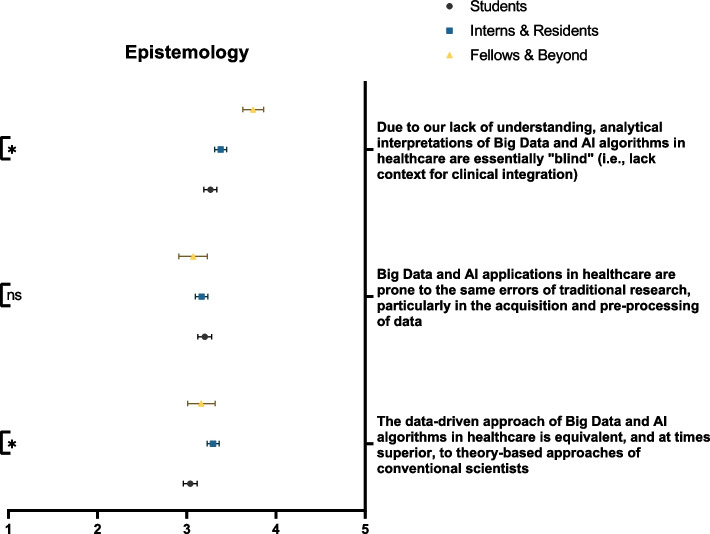
Participants’ awareness of the epistemology of Big Data and AI, stratified by educational rank. ns: not significant; * *p* < 0.05; ** *p* < 0.001

**Fig. 6:**
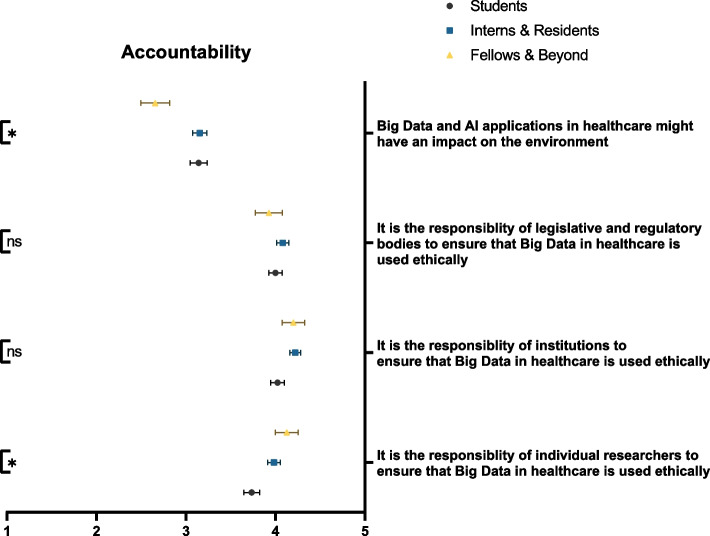
Participants’ awareness of the accountability associated with Big Data and AI applications in healthcare, stratified by educational rank. ns: not significant; * *p* < 0.05; ** *p* < 0.001

Respondents did not show clear-cut propensities when asked about the rigor of Big Data and AI methodology. The greatest proportion of participants believed that the methodologies of Big Data and AI were prone to the same errors as traditional research (39.9%). Moreover, participants agreed that the analytical interpretations of Big Data and AI algorithms lacked a proper context for clinical integration (42.3%). This notion was significantly more supported by senior physicians than both students and residents (*p* = 0.008) (Fig. 5). On the other hand, 37.6% of participants envisioned that the data-driven approaches associated with Big Data and AI were similar, if not more powerful, than the conventional theory-based approaches. It should be noted that with regards to Big Data and AI’s approach superiority, tendency for error, and utility within clinical research, participants often had neutral stances (38.4%, 32.2%, and 41.4%, respectively). On the other hand, there was an accord among respondents that the ethical use of Big Data and AI is a shared responsibility among researchers (67.6%), institutions (77.0%), and legislative bodies (72.1%). Only 36.3% of respondents recognized the impact of Big Data and AI on the environment. Such an impact was significantly more appreciated by students and residents than their more senior counterparts (*p* = 0.021) (Fig. 6). Table 2 demonstrates participants’ responses to knowledge sub-domains.

**Table 2:** Participants’ responses to knowledge sub-domains

Item	Disagree n (%)	Neutral n (%)	Agree n (%)
**Privacy & confidentiality**			
Big Data and AI applications in healthcare may predispose patients’ personal details (e.g., health information) to privacy breaches	114 (24.5%)	169 (36.3%)	183 (39.3%)
Under no circumstances, an in-house breaching of patients’ data might be necessary	207 (44.4%)	68 (14.6%)	191 (41.0%)
Big Data and AI applications in healthcare may predispose patient’s data to use by unauthorized personnel	107 (23.0%)	140 (30.0%)	219 (47.0%)
Patients’ data, embedded within Big Data and AI projects, could be used for alternative processes	74 (15.9%)	126 (27.0%)	266 (57.1%)
Ethical risks associated with Big Data and AI application in healthcare may be present across all steps of data management (e.g., collection, linking, and implementation)	62 (13.3%)	148 (31.8%)	256 (54.9%)
Linking data from different sources poses significant and novel ethical challenges	92 (19.7%)	152 (32.6%)	222 (47.6%)
**Informed consent**			
Designing and/or obtaining consent is an ethical limitation of Big Data and AI projects in healthcare	134 (28.8%)	138 (29.6%)	194 (41.6%)
Data usage permissions granted by informed consent must be determined by legislative authorities	49 (10.5%)	90 (19.3%)	327 (70.2%)
Obtaining consent for a broad range of future research projects not foreseen at the time of asking doesn’t qualify as “informed” consent	135 (29.0%)	123 (26.4%)	208 (44.6%)
The informed consent in Big Data and AI projects in healthcare lack transparency due to inherently complex inner-workings of novel AI algorithms	97 (20.8%)	166 (35.6%)	203 (43.6%)
**Ownership**			
In general, and in Big Data and AI projects in healthcare in particular, data, even at the individual-level, cannot be owned	92 (19.7%)	143 (30.7%)	231 (49.6%)
Parties conducting Big Data and AI projects in healthcare should be able to exert a quasi-control of patients’ data, as to market or to refrain from alienating intimate data’s core features, to protect data but also to participate in data-driven endeavors, and to use data for one’s own benefit or the benefit of others	46 (9.9%)	145 (31.1%)	275 (59.0%)
Under certain circumstances, data generated from Big Data and AI projects in healthcare could be utilized for marketization/commodification	231 (49.6%)	107 (23.0%)	128 (27.5%)
**Biases & divides**			
Big Data and AI application in healthcare could extend economic inequality	117 (25.1%)	190 (40.8%)	159 (34.1%)
Big Data and AI application in healthcare could promote health discrimination	166 (35.6%)	140 (30.0%)	160 (34.3%)
Big Data and AI models in healthcare have the inherent risk of augmenting the biases of their developers or the populations on which they were developed	85 (18.2%)	152 (32.6%)	229 (49.1%)
**Epistemology**			
The data-driven approach of Big Data and AI algorithms in healthcare is equivalent, and at times superior, to theory-based approaches of conventional scientists	112 (24.0%)	179 (38.4%)	175 (37.6%)
Big Data and AI application in healthcare is prone to the same errors of traditional research, particularly in the acquisition and pre-processing of data (e.g., checking data consistency)	130 (27.9%)	150 (32.2%)	186 (39.9%)
Due to our lack of understanding, analytical interpretations of Big Data and AI algorithms in healthcare are essentially “blind” (i.e., lack context for clinical integration)	76 (16.3%)	193 (41.4%)	197 (42.3%)
**Accountability**			
It is the responsibility of individual researchers to ensure that big data in healthcare is used ethically	59 (12.7%)	92 (19.7%)	315 (67.6%)
It is the responsibility of institutions to ensure that big data in healthcare is used ethically	32 (6.9%)	75 (16.1%)	359 (77.0%)
It is the responsibility of legislative and regulatory bodies to ensure that big data in healthcare is used ethically	37 (7.9%)	93 (20.0%)	336 (72.1%)
Big data and AI application in healthcare might have an impact on the environment	140 (30.0%)	157 (33.7%)	169 (36.3%)

### Attitudes

**Fig. 7:**
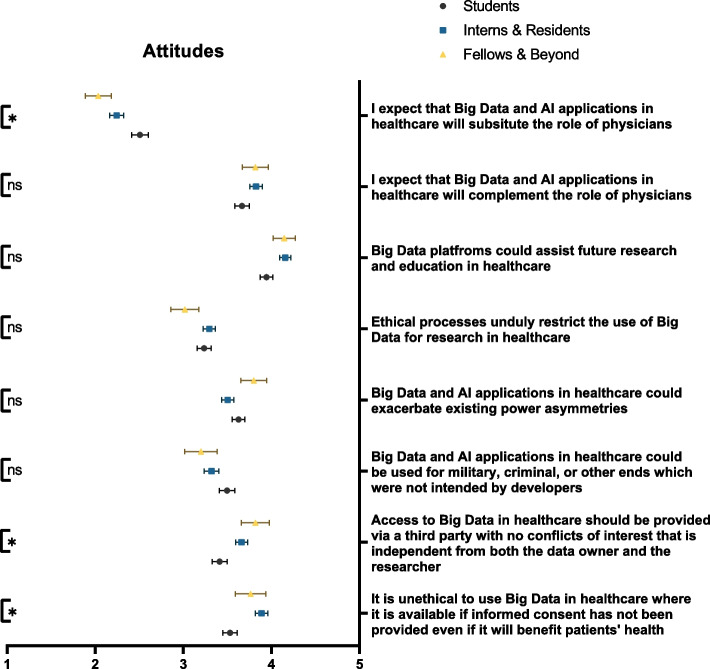
Participants’ attitudes towards the ethical dilemmas predisposed by Big Data and AI. ns: not significant; * *p* < 0.05; ** *p* < 0.001

Among our respondents, the overall view of the ethics of Big Data and AI were conflicting. While 57.5% believed that informed consent was a prerequisite to ethical Big Data implementation, 48.9% supported its unrestricted usage for military or criminal purposes. The former was significantly less acknowledged by students compared to residents (*p* = 0.006) (Fig. 7). With respect to the technology itself, respondents agreed that Big Data and AI are helpful within secondary healthcare practices (72.5%) and could complement the role of physicians (61.4%). Nonetheless, they were aware of its ability to exacerbate power asymmetries (52.8%). Table 3 demonstrates participants’ attitudes towards Big Data and AI.

**Table 3:** Participants’ attitudes and practices towards Big Data and AI

Item	Disagree n (%)	Neutral n (%)	Agree n (%)
**Attitudes**			
It is unethical to use Big Data in healthcare where it is available if informed consent has not been provided even if it will benefit patients’ health	61 (13.1%)	136 (29.2%)	269 (57.7%)
Access to Big Data in healthcare should be provided via a third party with no conflicts of interest that is independent both from the data owner and the researcher	62 (13.3%)	157 (33.7%)	247 (53.0%)
Big data and AI applications in healthcare could be used for military, criminal or other ends which were not intended by its developers	103 (22.1%)	135 (29.0%)	228 (48.9%)
Big Data and AI applications in healthcare could exacerbate existing power asymmetries by, for instance, giving a large amount of power to those already holding power over other people	55 (11.8%)	165 (35.4%)	246 (52.8%)
Ethical processes unduly restrict the use of Big Data for research in healthcare	104 (22.3%)	183 (39.3%)	179 (38.4%)
Big Data platform could assist future research and education in healthcare	29 (6.2%)	99 (21.2%)	338 (72.5%)
I expect Big Data and AI application in healthcare will complement the role of physicians	57 (12.2%)	123 (26.4%)	286 (61.4%)
I expect Big Data and AI application in healthcare will substitute the role of physicians	275 (59.0%)	108 (23.2%)	83 (17.8%)
**Practices**			
I have navigated the legal and regulatory aspects regarding the use of big data and AI applications in healthcare	244 (52.4%)	143 (30.7%)	79 (17.0%)
I have used AI-powered diagnostic tools in my practice	251 (53.9%)	116 (24.9%)	99 (21.2%)
Jordan has laws that regulate the use of AI and Big Data applications in healthcare practice	240 (51.5%)	159 (34.1%)	67 (14.4%)

### Practices

**Fig. 8:**
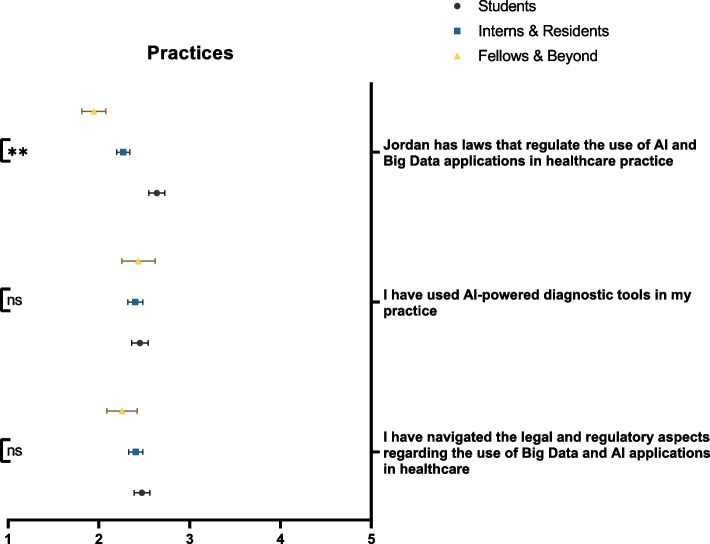
Participants’ current practices with regards to Big Data and AI. ns: not significant; * *p* < 0.05; ** *p* < 0.001

The majority of our included cohort reported no experiences using AI-powered tools (53.9%) and no exploration of the legal and regulatory landscapes associated with Big Data and AI applications in healthcare (52.4%). Moreover, most respondents believed that Jordan does not have the capacity to regulate such technology (51.5%). Figure 8 demonstrates participants’ practices stratified by educational rank.

### Institutional differences in Big Data and AI ethics perception of respondents

Compared to public, private, and university institutions, respondents from military hospitals were significantly more likely to support in-house breaching of patient data (*p* = 0.001). In fact, respondents from military hospitals and KHCC were significantly more likely to support the idea of quasi-control over patients’ data (*p* < 0.001). Interestingly, compared to university hospitals, respondents from private institutions were significantly more supportive of the notion that data cannot be owned (*p* = 0.009) and that Big Data and AI interpretations are of limited value within a real clinical context (*p* = 0.005). Moreover, respondents from private institutions were significantly more aware of the need for informed consent for the ethical use of Big Data (*p* = 0.005), and the ability of such technologies to create power asymmetries (*p* = 0.029). Finally, respondents from KHCC were more appreciative of the potential role of Big Data and AI in shaping education and research compared to university hospitals (*p* = 0.011).

## Discussion

### Summary of findings

Our examination of a subset of Jordanian healthcare workers, including medical students, demonstrated a fair, yet limited, awareness of the ethical and moral dilemmas associated with Big Data and AI; at least on the superficial level across many themes, namely, privacy and confidentiality, informed consent, ownership, biases and divides, epistemology, and accountability. Participants were appreciative of the following: Big Data and AI are associated with privacy risks across all stages of implementation, obtaining informed consent is an ethical limitation of the technology and cannot be trusted due to the complex inner working of algorithms, ethical inappropriateness of “broad consent”, inability to own data, Big Data and AI are associated with inherent biases precipitated by their development process, and vulnerability of such technology to the same errors of conventional research and its lack of an appropriate clinical context for integration. Furthermore, while the attitudes of participants were generally positive, they believed that Jordan does not have legal capacity to regulate Big Data and AI.

### Content within the literature

The utilization of Big Data and AI in healthcare has offered numerous possibilities that hold the potential to improve patient care and healthcare systems' efficiency. However, this integration also brings a range of significant privacy risks that demand careful consideration. One of the most concerning privacy risks is the potential for data breaches and unauthorized access to patient-sensitive information. Despite the implementation of security measures, a critical privacy risk comes from the process of de-identification, which, paradoxically, introduces its own set of vulnerabilities.

One of the primary concerns is the potential for re-identification, where, even after extensive de-identification efforts, health data remains vulnerable to re-identification breaches. In their study, Patsakis et al. demonstrate how Large Language Models (LLM) were found to have a devastating impact on document deanonymization. Even when not explicitly trained for this purpose, LLMs can use minor knowledge hints to achieve complete deanonymization of data [25]. Moreover, organizations are increasingly likely to use LLMs to gain visibility about their customers’ data and, more concerning, trends. This sheds light on the threat LLM poses in the era of Big Data and AI. On another note, El Emam et al. demonstrated that even after immense de-identification efforts, re-identification is a plausible risk [26]. Fredrikson et al. show that through inference attacks, the process by which AI algorithms uncover sensitive information from what was assumed to be non-sensitive data, re-identification could be achieved [27]. Thus, researchers and scientists should be proficient in de-identifying data per the most appropriate guidelines. Also, it is essential to establish complementary legal safeguards and governance standards such as data-sharing agreements for the aims of prohibiting re-identification attempts and delineating accountability.

Informed consent has always been the cornerstone of medical ethics, emphasizing on the importance of providing patients with information about their proposed treatments to enable autonomous decision-making [28]. However, with the integration of Big Data or AI systems that are able to make predictions or find trends within data, secondary uses of data become apparent and the concept of ‘consent’ is challenged [7]. In the context of Big Data, informed consent's limitations become apparent, and broad consent emerges as a potential solution to navigate the complexities of healthcare data sharing and research, while respecting patient privacy concerns [11]. However, this solution, although can provide legal coverage for Big Data or AI applications, is not ethical, to say the least. Within broad consent, neither the researcher nor patient knows what data or even usage objectives of data are to be conducted, since these objectives are often determined at a future timeframe when the data is mature.

In addition to the ethical considerations, the legal aspect of data ownership is a subject of ongoing debate and ambiguity. The implementation of recent Big Data and AI tools has significantly increased not only the importance of owning this data, but also increased its value to both public and private health sectors [29]. Individual ownership of data, including healthcare data, is contrary to well-established legal precedents in the United States, the United Kingdom, and many other jurisdictions [30]. This perspective is due to the long-established legal model that does not recognize property interests in facts or information. In contrast, European data protection regulations, typically frame data-related rights as an extension of fundamental human rights, which gives individuals a certain degree of control over their data and also implies that they are unsuitable for commodification or commercialization. Beyond European and American perspectives, various national legal frameworks differ considerably in their stance on data ownership. Contract law plays a significant role in defining the rights of data originators and processors; however, it does not address the foundational question of who owns the data [5].

Big Data and AI offer promising solutions for global healthcare challenges, addressing resource shortages and improving healthcare infrastructure [31, 32]. However, this transformative potential also carries the substantial risks of exacerbating the already existing health and economic inequalities. If not carefully and thoughtfully adopted, AI may unintentionally reinforce existing disparities among various demographic groups. Algorithms that are not rigorously tested across diverse demographics can yield inaccurate results, leading to diagnostic tests that perform better for some groups at the expense of others [33]. An example of this bias emerged when an AI-powered dermatology application, trained predominantly on Caucasian skin types, showed reduced diagnostic accuracy for black patients [34]. This implies that such an AI-powered tool, despite its diagnostic accuracy, may be of limited use in areas such as Asia, Sub-Saharan Africa, Latin America, or even the Middle East due to the different epidemiology of skin diseases and the fact an ML model cannot explain any phenomenon beyond its trained dataset. The latter is of utmost importance as it can augment already existing disparities. For example, ML algorithms inherently exhibit bias against underprivileged and minority populations as those have lesser access to healthcare services; thus, fewer data points [35]. This was exemplified by Jacoba et al., who demonstrated that AI-powered diagnostic tools for retinal diseases may show reduced accuracy in underrepresented populations due to the lack of accessible representative images for 45% of the global population [36].

Big Data and AI applications introduce a variety of epistemological challenges, mainly that of data collection and data analysis. In terms of data collection, researchers must take extremely cautious steps when acquiring and preprocessing data. Such is the case due to the fact that most generated datasets utilized in Big Data are not the output of valid and reliable tools [37]. On the analytic front, analysis within Big Data and AI is entirely data-driven; an approach which is seen to produce irreproducible studies, unreliable data, and utilizes inappropriate statistics by anti-data fundamentalists [32, 38]. While such an approach is more precise than traditional theory-based science, its processes of extracting and deriving meaning from even hidden trends are semantically blind [37]. Nonetheless, recent epistemological literature considers data and theory-driven approaches as complementary approaches that are potentially convergent rather than radically divergent [1].

Within the Jordanian landscape, Big Data and AI applications are gaining traction. In our study, we demonstrated the knowledge, attitudes, and practices of Jordanian healthcare workers, including medical students, toward Big Data and AI. In terms of risk, participants were moderately aware of the impact of Big Data and AI on privacy, consent, and extending inequality. Nonetheless, some items may mirror ideological and cultural differences compared to Western standards. For example, 59% of participants justify an in-house data breach under certain scenarios. The Apple-FBI dispute in 2016 clearly showcased that the aforementioned notion could never be accepted in the West, particularly the United States [39]. Another example is the dominant stance for institutions to have a quasi-control of patients’ data. This may show that Jordanian institutions or their employed healthcare workers are willing to use their patients’ data for commodification purposes if the opportunity allows. Nonetheless, due to the significant lack of experience with respect to Big Data and AI, participants' poor awareness of epistemological or methodological biases associated with such technology might be justified. In fact, healthcare workers are not confident that even the Jordanian healthcare landscape could adopt or regulate such technology.

### Limitations

Our study represents a preliminary investigation into the understanding of ethical risks associated with Big Data and AI. However, it is bound by a number of limitations which include the use of a face-validated questionnaire, vulnerability to biases introduced by cross-sectional designs, the close-ended nature of the questionnaire, and a convenient sampling technique. The latter is particularly important as it may fail to produce a sample representative of the Jordanian healthcare workforce. Moreover, the full spectrum of psychometric properties of the developed scale was not calculated. However, the questionnaire was not designed to produce scores for its sub-components nor are there similar tools to test its validity against.

## Conclusion

In short, Jordanian healthcare workers, representing both under- and post graduate participants from different institutions, have limited awareness of the ethical risks and principles associated with Big Data and AI. Ultimately, such levels of awareness may predispose patients’ data to unwarranted breaches, allure institutions to adopt or implement AI models with limited clinical value, and foster poor data handling practices across both clinical and research practices. Therefore, we recommend that medical institutions strive to develop, adopt, and promote awareness towards the clinical utility of Big Data and AI and its associated ethical risks. Secondly, institutions should also be encouraged to draft ethical policies that adhere to universal ethical principles and are in line with the institutions’ resources for Big Data and AI adoption. Finally, policy makers should anticipate the upsurge of Big Data and AI technology by drafting regulatory laws which aim to regulate the development, implementation, and monetization of Big Data and AI technology.

## Supplementary Information

**Additional file 1.**

**Additional file 2: Supplementary Table 1. **This tables demonstrates the p-values of the chi-square tests, which corresponding to differences in responses among selected groups.

## Data Availability

The datasets used and/or analyzed during the current study are available from the corresponding author on reasonable request.
