# Correction to: Evaluating the understanding of the ethical and moral challenges of Big Data and AI among Jordanian medical students, physicians in training, and senior practitioners: a cross-sectional study

**DOI:** 10.1186/s12910-024-01027-x

**Published:** 2024-03-05

**Authors:** Abdallah Al-Ani, Abdallah Rayyan, Ahmad Maswadeh, Hala Sultan, Ahmed Alhammouri, Hadeel Asfour, Tariq Alrawajih, Sarah Al Sharie, Fahed Al Karmi, Ahmed Mahmoud Al-Azzam, Asem Mansour, Maysa Al-Hussaini

**Affiliations:** 1 Office of Scientific Affairs and Research, King Hussein Cancer Center, Amman, Jordan; 2 Faculty of Medicine, University of Jordan, Amman, Jordan; 3 Faculty of Medicine, Yarmouk University, Irbid, Jordan; 4 Office of Director General, King Hussein Cancer Center, Amman, Jordan; 5 Department of Pathology and Laboratory Medicine, King Hussein Cancer Center, 202 Queen Rania Street, Amman, 11941 Jordan

**Correction to: BMC Medical Ethics (2024) 25:1**

10.1186/s12910-024-01008-0

Following publication of the original article [1], author’s name Ahmed Mahmoud Al-Azzam was incorrectly written as Ahmad Azzam.

The original article has been corrected.
