# Correction: Evaluating the understanding of the ethical and moral challenges of Big Data and AI among Jordanian medical students, physicians in training, and senior practitioners: a cross-sectional study

**DOI:** 10.1186/s12910-025-01184-7

**Published:** 2025-02-25

**Authors:** Abdallah Al-Ani, Abdallah Rayyan, Ahmad Maswadeh, Hala Sultan, Ahmed Alhammouri, Hadeel Asfour, Tariq Alrawajih, Sarah Al Sharie, Fahed Al Karmi, Ahmed Mahmoud Al-Azzam, Asem Mansour, Maysa Al-Hussaini

**Affiliations:** 1 Office of Scientific Affairs and Research, King Hussein Cancer Center, Amman, Jordan; 2 Faculty of Medicine, University of Jordan, Amman, Jordan; 3 Faculty of Medicine, Yarmouk University, Irbid, Jordan; 4 Office of Director General, King Hussein Cancer Center, Amman, Jordan; 5 Department of Pathology and Laboratory Medicine, King Hussein Cancer Center, 202 Queen Rania Street, Amman, 11941 Jordan

**Correction: BMC Med Ethics 25, 18 (2024)**

**https://doi.org/10.1186/s12910-024-01008-0**

In the original publication of this article [1], there was an error in the authors names, Ahmed Alhammouri and Ahmed Mahmoud Al-Azzam. The incorrect and correct authors names is listed in this correction article; the original article has been updated.

Incorrect Author Names: Ahmad Alhammouri and Ahmad Azzam

Correct Author Names: Ahmed Alhammouri and Ahmed Mahmoud Al-Azzam 
