# Supplementary material for: Evaluating the understanding of the ethical and moral challenges of Big Data and AI among Jordanian medical students, physicians in training, and senior practitioners: a cross-sectional study
Source: BMC Med Ethics. 2024 Feb 17;25:18. doi: 10.1186/s12910-024-01008-0 (PMC10873950; doi:10.1186/s12910-024-01008-0)
Supplement: Supplementary file 1 — Additional file 1. [file 12910_2024_1008_MOESM1_ESM.pdf]

Evaluating the understanding of the ethical and moral challenges of Big Data and AI among Jordanian medical students, physicians in training, and senior practitioners: A cross-sectional study

1. Demographics

____

2. Age

____

3. Gender
   - Male
   - Female

4. Education Level
   - 4th year medical student
   - 5th year medical student
   - 6th year medical student
   - Intern
   - Resident
   - Fellow
   - Specialist
   - Consultant/ Professor

5. Number of Published Papers

____

6. Current Institution
   - University Hospital
   - Public Sector
   - Private Sector
   - Royal Medical Services
   - KHCC

7. I am familiar with Big Data and AI applications in healthcare
   - Strongly Disagree
   - Disagree
   - Neutral
   - Agree
   - Strongly Agree

DRAFT

Please read the following information before proceeding to answer the rest of the questionnaire.

Based on widely accepted industry definitions and understanding, ***Big Data*** *refers to extremely large and complex data sets that cannot be easily processed or analyzed using traditional data processing methods. These data sets often come from a variety of sources, such as social media, sensor data, and online transactions, and can be used to uncover patterns and insights that can inform business decisions and inform research.* ***AI, or artificial intelligence****, is a broad field of study that involves the development of computer systems and algorithms that can perform tasks that typically require human intelligence, such as visual perception, speech recognition, decision-making, and language understanding.*

Here are some examples for Big Data and AI applications in healthcare and research:

1. ***Medical Chatbot and Virtual Assistants****: AI-powered chatbots and virtual assistants are being used to provide patients with quick and accurate medical information and even help them schedule appointments and refill prescriptions.*
2. ***Robotics and Automation****: AI is being used to automate repetitive tasks in hospitals and clinics, such as scheduling appointments, managing patient records, and even performing basic medical procedures.*
3. ***Medical Imaging****: AI-powered algorithms can be used to analyze medical images, such as CT and MRI scans, to identify potential issues and aid in diagnosis.*
4. ***Personalized Medicine****: By analyzing large amounts of genetic data, researchers can develop more targeted and effective treatments for individual patients.*

DRAFT

Privacy and Confidentiality
In the context of data, privacy refers to the right of individuals to control how their personal information is collected, used, and shared. Confidentiality, on the other hand, refers to measures taken to protect sensitive information from unauthorized access or disclosure.

Knowledge

1. Big Data and AI applications in healthcare may predispose patients' personal details (e.g., health information) to privacy breaches
   - o Strongly Disagree
   - o Disagree
   - o Neutral
   - o Agree
   - o Strongly Agree

2. Under no circumstances, should the breaching of patients' data be permitted
   - o Strongly Disagree

- Disagree
- Neutral
- Agree
- Strongly Agree

3. Big Data and AI applications in healthcare may predispose patient's data to use by unauthorized personnel
    - Strongly Disagree
    - Disagree
    - Neutral
    - Agree
    - Strongly Agree

4. Patients' data, included in Big Data and AI projects, could be used for alternative processes
    - Strongly Disagree
    - Disagree
    - Neutral
    - Agree
    - Strongly Agree

5. Ethical risks associated with Big Data and AI application in healthcare may be present across all steps of data management (e.g., collection, linking, and implementation)
    - Strongly Disagree
    - Disagree
    - Neutral
    - Agree
    - Strongly Agree

6. Linking data from different sources poses significant and novel ethical challenges
    - Strongly Disagree
    - Disagree
    - Neutral
    - Agree
    - Strongly Agree

DRAFT

Informed Consent

In the context of data, informed consent is a process that helps ensure that a person is fully informed of the implications, risks, and benefits of a proposed study, research or treatment and that they voluntarily agree to participate after being given enough information to make an informed decision.

7. Designing and/or obtaining consent is an ethical limitation of Big Data and AI projects in healthcare
    - Strongly Disagree
    - Disagree
    - Neutral
    - Agree
    - Strongly Agree

8. Data usage permissions granted by informed consent must be determined by legal authorities
    - Strongly Disagree
    - Disagree
    - Neutral
    - Agree
    - Strongly Agree

9. Obtaining consent for a broad range of future research projects not planned at the time of asking does not qualify as "informed" consent
    - Strongly Disagree
    - Disagree
    - Neutral
    - Agree
    - Strongly Agree

10. The informed consent in Big Data and AI projects in healthcare lack transparency due to inherently complex inner-workings of novel AI algorithms
    - Strongly Disagree
    - Disagree
    - Neutral
    - Agree
    - Strongly Agree

Ownership

In the context of data, ownership refers to the legal right to possess, control, and use a particular set of information, it can be held by individuals, organizations, or governments and it can be acquired and lost through different means.

11. In general, and in Big Data and AI projects in healthcare in particular, data, even at the individual-level, cannot be owned
    - Strongly Disagree
    - Disagree
    - Neutral
    - Agree
    - Strongly Agree

12. Parties conducting Big Data and AI projects in healthcare should be able to apply a quasi-control of patients' data, as to market or refrain from

DRAFT

alienating intimate data’s core features, to protect data but also to participate in data-driven endeavors, and use data for one’s own benefit or the benefit of others

- Strongly Disagree
- Disagree
- Neutral
- Agree
- Strongly Agree

13. Under certain circumstances, data generated from Big Data and AI projects in healthcare could be utilized for marketization
    - Strongly Disagree
    - Disagree
    - Neutral
    - Agree
    - Strongly Agree

Biases and Divides

In the context of data, biases and divides refer to the ways in which data can be skewed, distorted, or incomplete in ways that perpetuate or amplify existing inequalities or disparities in society. Data bias occurs when a dataset used for analysis or decision-making is not representative of the population it intends to describe and data divide refers to the gaps in data availability and quality between different population groups or geographic areas.

14. Big Data and AI application in healthcare could extend economic inequality
    - Strongly Disagree
    - Disagree
    - Neutral
    - Agree
    - Strongly Agree

15. Big Data and AI application in healthcare could promote health discrimination
    - Strongly Disagree
    - Disagree
    - Neutral
    - Agree
    - Strongly Agree

16. Big Data and AI models in healthcare have the inherent risk of augmenting the biases of their developers or the populations on which they were developed
    - Strongly Disagree
    - Disagree
    - Neutral
    - Agree
    - Strongly Agree

Epistemology

DRAFT

In the context of data, epistemology refers to the branch of philosophy that deals with the nature of knowledge and how it is acquired. It concerns questions such as how we know what we know, what counts as knowledge, and how we can be certain that our knowledge is true. Addressing epistemological questions is crucial for understanding the limitations and potential of data-based knowledge and also helps to ensure that data-driven decisions are well-informed, accurate and fair.

17. The data-driven approach of Big Data and AI algorithms in healthcare is equivalent, and at times superior, to theory-based approaches of conventional scientists
    - Strongly Disagree
    - Disagree
    - Neutral
    - Agree
    - Strongly Agree

18. Big Data and AI application in healthcare is prone to the same errors of traditional research, particularly in the acquisition and pre-processing of data (e.g., checking data consistency)
    - Strongly Disagree
    - Disagree
    - Neutral
    - Agree
    - Strongly Agree

19. Due to our lack of understanding, analytical interpretations of Big Data and AI algorithms in healthcare are essentially "blind" (i.e., lack context for clinical integration)
    - Strongly Disagree
    - Disagree
    - Neutral
    - Agree
    - Strongly Agree

Accountability

In the context of data, accountability refers to the responsibility and obligation of individuals, organizations, or governments to account for their actions related to the collection, use, and management of data. This includes ensuring that data is used ethically and legally, that it is accurate and reliable, and that it is protected from unauthorized access or misuse. It also implies the need for transparency, governance, and oversight in the data life-cycle management and compliance with data protection and privacy laws.

20. It is the responsibility of individual researchers to ensure that big data in healthcare is used ethically
    - Strongly Disagree
    - Disagree
    - Neutral
    - Agree
    - Strongly Agree

DRAFT

21. It is the responsibility of institutions to ensure that big data in healthcare is used ethically
    - Strongly Disagree
    - Disagree
    - Neutral
    - Agree
    - Strongly Agree

22. It is the responsibility of llegislative and regulatory bodies to ensure that big data in healthcare is used ethically
    - Strongly Disagree
    - Disagree
    - Neutral
    - Agree
    - Strongly Agree

23. Big data and AI application in healthcare might have an impact on the environment
    - Strongly Disagree
    - Disagree
    - Neutral
    - Agree
    - Strongly Agree

Attitude

24. It is unethical to use Big Data in healthcare where it is available if informed consent has not been provided even if it will benefit patients' health
    - Strongly Disagree
    - Disagree
    - Neutral
    - Agree
    - Strongly Agree

25. Access to Big Data in healthcare should be provided via a third party with no conflicts of interest that is independent both from the data owner and the researcher
    - Strongly Disagree
    - Disagree
    - Neutral
    - Agree
    - Strongly Agree

26. Big Data and AI applications in healthcare could exacerbate existing power asymmetries by, for instance, giving a large amount of power to those already holding power over other people
    - Strongly Disagree
    - Disagree
    - Neutral

DRAFT

- Agree
- Strongly Agree

27. Ethical processes unduly restrict the use of Big Data for research in healthcare
    - Strongly Disagree
    - Disagree
    - Neutral
    - Agree
    - Strongly Agree

28. Big Data platform could assist future research and education in healthcare
    - Strongly Disagree
    - Disagree
    - Neutral
    - Agree
    - Strongly Agree

29. I expect Big Data and AI application in healthcare will complement the role of physicians
    - Strongly Disagree
    - Disagree
    - Neutral
    - Agree
    - Strongly Agree

30. I expect Big Data and AI application in healthcare will substitute the role of physicians
    - Strongly Disagree
    - Disagree
    - Neutral
    - Agree
    - Strongly Agree

Practice

31. I have navigated the legal and regulatory aspects regardi ng the use of big data and AI applications in healthcare
    - Strongly Disagree
    - Disagree
    - Neutral
    - Agree
    - Strongly Agree

32. I have used AI-powered diagnostic tools in my practice
    - Strongly Disagree
    - Disagree

DRAFT

- Neutral
- Agree
- Strongly Agree

33. Jordan has laws that regulate the use of AI and Big Data applications in healthcare practice
    - Strongly Disagree
    - Disagree
    - Neutral
    - Agree
    - Strongly Agree

34. Please feel free to share any comments on the topic or questionnaire below
____
____
____

DRAFT
