# Supplementary material for: Evaluating the understanding of the ethical and moral challenges of Big Data and AI among Jordanian medical students, physicians in training, and senior practitioners: a cross-sectional study
Source: BMC Med Ethics. 2024 Feb 17;25:18. doi: 10.1186/s12910-024-01008-0 (PMC10873950; doi:10.1186/s12910-024-01008-0)
Supplement: Supplementary file 2 — Additional file 2: Supplementary Table 1. This tables demonstrates the p-values of the chi-square tests, which corresponding to differences in responses among selected groups. [file 12910_2024_1008_MOESM2_ESM.pdf]

Evaluating the understanding of the ethical and moral challenges of Big Data and AI among Jordanian medical students, physicians in training, and senior practitioners: A cross-sectional study

Supplementary Table 1: This tables demonstrates the p-values of the chi-square tests, which corresponding to differences in responses among selected groups.

Item	Gender	Level of education	Publication status
**Privacy & confidentiality**			
Big Data and AI applications in healthcare may predispose patients' personal details (e.g., health information) to privacy breaches	0.966	0.422	0.585
Under no circumstances, an in-house breaching of patients' data might be necessary	0.420	**0.044**	0.985
Big Data and AI applications in healthcare may predispose patients' data to use by unauthorized personnel	0.841	**0.005**	0.670
Patients' data, embedded within Big Data and AI projects, could be used for alternative processes	0.934	**0.007**	**0.007**
Ethical risks associated with Big Data and AI applications in healthcare may be present across all steps of data management (e.g., collection, linking, and implementation)	0.179	0.062	0.893
Linking data from different sources poses significant and novel ethical challenges	0.278	0.844	0.822
**Informed consent**			
Designing and/or obtaining consent is an ethical limitation of Big Data and AI projects in healthcare	0.624	0.092	0.088
Data usage permissions granted by informed consent must be determined by legislative authorities	0.366	**0.017**	0.350
Obtaining consent for a broad range of future research projects not foreseen at the time of asking doesn't qualify as "informed" consent	0.874	**0.036**	0.363
The informed consent in Big Data and AI projects in healthcare lack transparency due to inherently complex inner-workings of novel AI algorithms	0.951	0.156	0.463
**Ownership**			
In general, and in Big Data and AI projects in healthcare in particular, data, even at the individual-level, cannot be owned	0.293	0.072	0.077
Parties conducting Big Data and AI projects in healthcare should be able to exert a quasi-control of patients' data, as to market or to refrain from alienating intimate data's core features, to protect data but also to participate in data-driven endeavors, and to use data for one's own benefit or the benefit of others	**0.028**	**0.002**	**0.005**
Under certain circumstances, data generated from Big Data and AI projects in healthcare could be utilized for marketization/commodification	0.385	**0.011**	0.775
**Biases & divides**			
Big Data and AI application in healthcare could extend economic inequality	0.743	0.155	0.811
Big Data and AI application in healthcare could promote health discrimination	0.850	0.181	0.310
Big Data and AI models in healthcare have the inherent risk of augmenting the biases of their developers or the populations on which they were developed	0.324	0.245	0.896
**Epistemology**			
The data-driven approach of Big Data and AI algorithms in healthcare is equivalent, and at times superior, to theory-based approaches of	0.231	0.260	**0.000**

conventional scientists			
Big Data and AI application in healthcare is prone to the same errors of traditional research, particularly in the acquisition and pre-processing of data (e.g., checking data consistency)	0.084	0.296	0.710
Due to our lack of understanding, analytical interpretations of Big Data and AI algorithms in healthcare are essentially “blind” (i.e., lack context for clinical integration)	0.155	**0.012**	0.111
**Accountability**			
It is the responsibility of individual researchers to ensure that big data in healthcare is used ethically	0.185	**0.014**	0.313
It is the responsibility of institutions to ensure that big data in healthcare is used ethically	0.088	0.231	0.081
It is the responsibility of legislative and regulatory bodies to ensure that big data in healthcare is used ethically	**0.033**	0.299	0.318
Big data and AI application in healthcare might have an impact on the environment	0.880	0.072	0.102
**Attitudes**			
It is unethical to use Big Data in healthcare where it is available if informed consent has not been provided even if it will benefit patients’ health	0.555	0.553	0.745
Access to Big Data in healthcare should be provided via a third party with no conflicts of interest that is independent both from the data owner and the researcher	0.602	0.142	0.225
Big data and AI applications in healthcare could be used for military, criminal or other ends which were not intended by its developers	0.197	0.534	0.791
Big Data and AI applications in healthcare could exacerbate existing power asymmetries by, for instance, giving a large amount of power to those already holding power over other people	0.615	0.453	0.548
Ethical processes unduly restrict the use of Big Data for research in healthcare	**0.048**	0.958	0.996
Big Data platforms could assist future research and education in healthcare	0.131	0.715	0.414
I expect that Big Data and AI application in healthcare will complement the role of physicians	0.401	0.232	0.063
I expect that Big Data and AI application in healthcare will substitute the role of physicians	**0.000**	0.198	0.064
**Practices**			
I have navigated the legal and regulatory aspects regarding the use of big data and AI applications in healthcare	**0.030**	0.336	0.450
I have used AI-powered diagnostic tools in my practice	**0.013**	0.453	0.354
Jordan has laws that regulate the use of AI and Big Data applications in healthcare practice	0.854	**0.003**	0.153
